# Formulation of moment equations for rarefied gases within two frameworks of non-equilibrium thermodynamics: RET and GENERIC

**DOI:** 10.1098/rsta.2019.0174

**Published:** 2020-03-30

**Authors:** Hans Christian Öttinger, Henning Struchtrup, Manuel Torrilhon

**Affiliations:** 1 Department of Materials, ETH Zürich, HCP F 47.2, 8093 Zürich, Switzerland; 2 Department of Mechanical Engineering, University of Victoria, Victoria, British Columbia, Canada V8W 2Y2; 3 Center for Computational Engineering Sciences, RWTH Aachen, 52062 Aachen, Germany

**Keywords:** rational extended thermodynamics, GENERIC, Boltzmann equation, moment equations, closure, Onsager–Casimir symmetry

## Abstract

In this work, we make a further step in bringing together different approaches to non-equilibrium thermodynamics. The structure of the moment hierarchy derived from the Boltzmann equation is at the heart of rational extended thermodynamics (RET, developed by Ingo Müller and Tommaso Ruggeri). Whereas the full moment hierarchy has the structure expressed in the general equation for the nonequilibrium reversible–irreversible coup- ling (GENERIC), the Poisson bracket structure of reversible dynamics postulated in that approach is a major obstacle for truncating moment hierarchies, which seems to work only in exceptional cases (most importantly, for the five moments associated with conservation laws). The practical importance of truncated moment hierarchies in rarefied gas dynamics and microfluidics motivates us to develop a new strategy for establishing the full GENERIC structure of truncated moment equations, based on non-entropy-producing irreversible processes associated with Casimir symmetry. Detailed results are given for the special case of 10 moments.

This article is part of the theme issue ‘Fundamental aspects of nonequilibrium thermodynamics’.

## 1. Introduction

The purpose of nonequilibrium thermodynamics is to recognize ‘good’ equations. From a physical perspective, this means that thermodynamically admissible evolution equations should guarantee proper balance equations for energy and entropy. From a mathematical perspective, this means that nonequilibrium thermodynamics should provide sufficient conditions for proving the existence and uniqueness of solutions of evolution equations (whenever physically appropriate).

There exists a large number of frameworks of nonequilibrium thermodynamics developed by various groups working in different fields (mostly in physics and chemical engineering). In the last 20 years, nonequilibrium thermodynamics has progressed significantly by clarifying the relations between different frameworks, allowing us to recognize the common principles and the most versatile approaches. A coherent appearance of the field is essential for the acceptance of nonequilibrium thermodynamics as a useful tool in applications. The purpose of this paper is to investigate the relationship between rational extended thermodynamics (RET) and the general equation for the nonequilibrium reversible–irreversible coupling (GENERIC). A common feature of these two frameworks is that they both are underpinned by statistical mechanics (see [1,2] for GENERIC and §2 and references therein for RET).

Any framework of nonequilibrium thermodynamics has to contain the well-established theories of equilibrium thermodynamics and linear irreversible thermodynamics [3] as limiting cases. In particular, linear irreversible thermodynamics includes Onsager–Casimir symmetry [4–6], which is a consequence of the behaviour of the evolution equations under time reversal. We here restrict ourselves to thermodynamic frameworks based on differential evolution equations for extended lists of variables (rather than memory functionals). Finding a list of variables that allows us to formulate an autonomous set of evolution equations is a challenging first step in any application of nonequilibrium thermodynamics. This step should be appreciated as an expression of deep insight, not as an annoying closure problem [7]. Extended thermodynamics has been developed since the 1960s, starting with pioneering work by Müller [8]. Originally, the motivation for extended thermodynamics came from the moment method for the Boltzmann equation in the kinetic theory of gases, and most applications were for gases. In the early work on extended thermodynamics, the fluxes of conserved quantities have been chosen as additional variables, and also fluxes of fluxes and even higher fluxes. Over time, two schools have emerged (see the article by Jou in this theme issue), nowadays known as rational extended thermodynamics (RET) [9] and extended irreversible thermodynamics (EIT) [10].

The restriction to the conserved quantities and fluxes appearing in balance equations as variables is motivated by the fact that their convection behaviour is well-understood so that one can focus entirely on the irreversible processes, which are usually associated with entropy production. Convection conserves entropy, and guaranteeing the non-negativity of entropy production by irreversible processes is a central element of any framework of nonequilibrium thermodynamics.

The use of more general structural variables became possible by the generalization of convection in terms of Hamiltonian dynamics with degenerate Poisson brackets. Nonequilibrium thermodynamics based on Poisson and dissipative brackets has been developed since the 1980s, starting with pioneering work by Grmela [11,12]. The so-called bracket formalism uses a single generator (a free energy) and two brackets for generating reversible and irreversible dynamics [13], whereas the GENERIC framework uses two generators (energy and entropy) and two brackets [14,15]. These two frameworks have been compared in detail in [16–19]. The conclusion is that ‘whereas as far as macroscopic systems are concerned in all the investigated systems so far a complete agreement between the one and two-generator formalisms is found, for microscopic systems [considered on the level of configurational distribution functions] small but significant differences exist with the existing evidence (by comparison to other theories) in favour of the two-generator formalism’ [19].

Comparisons of GENERIC with EIT have been offered in Sect. V.A of [20] and Sect. 5.1.6 of [14]. A detailed comparison with a version of rational thermodynamics [21], in which Liu’s procedure [22] for guaranteeing the non-negativity of entropy production is used, has been made in the context of a simple discrete system [23].

## 2. Kinetic theory of rarefied gases

Before we present and compare two thermodynamic frameworks, RET and GENERIC, we consider the kinetic theory of rarefied gases. In the subsequent developments, we need the moment expansion obtained from the Boltzmann equation for rarefied gases for two reasons: (i) the formulation of RET is guided by the structure of the moment expansion, and (ii) the 10 moment expansion serves as our specific example for a detailed comparison of RET and GENERIC.

### (a) Boltzmann equation

The basic quantity in kinetic theory is the particle distribution function *f*(**r**, *t*, **c**), where **r** and *t* are the space and time variables, respectively, and **c** denotes the microscopic velocities of particles. The distribution function is defined such that *f*(**r**, *t*, **c**) *d***c***d***r** gives the number of gas particles in the phase space cell *d***c***d***r** at time *t*. The space–time evolution of the distribution function is determined by the Boltzmann equation

2.1 $$\frac{\partial f}{\partial t}+c_{k}\frac{\partial f}{\partial r_{k}}=S(f,f),$$

where S(f,f) denotes the Boltzmann collision term. For our purposes, it is sufficient to list its most important properties (the full expression for S can be found in the literature [24–26]): Mass, momentum and energy are conserved in a collision, the production of entropy is always nonnegative (H-theorem), and in equilibrium the phase density is a Maxwellian distribution, i.e.

2.2 $$S=0⟹f=f_{\text{M}}=\frac{\rho }{m}\frac{1}{{\sqrt{2\pi \theta }}^{3}}\exp\left[-\frac{C^{2}}{2\theta }\right],$$

where *m* is the mass of a gas particle, *ρ* is the mass density, *θ* = *k*_B_*T*/*m* is the temperature in energy units, *k*_B_ is the Boltzmann constant, **C** = **c − *v*** is the peculiar velocity and **v** is the centre of mass velocity. Owing to the details of the collision term, the Boltzmann equation is a nonlinear integro differential equation for *f*. Its theory and solution are discussed in a wide body of literature, e.g. [25,26]. Also, the laws of classical hydrodynamics—the Navier–Stokes and Fourier equations—can be derived in the collision dominated regime, i.e. when the ratio of the mean free path of the particles and the length scale of the process, known as the Knudsen number, is sufficiently small [24–26].

Typically, one is not interested in the full details of the distribution function, but rather in some physically relevant averages, in particular mass density *ρ*, momentum density **M** and energy density *ϵ*, which are given by

2.3 $$\rho =m\int fdc,\;M_{i}=\rho v_{i}=m\int c_{i}fdc,\;\epsilon =\frac{m}{2}\int c^{2}fdc.$$

General moments are symmetric tensors of the form

2.4 $$F_{i_{1}i_{2}\cdots i_{n}}=m\int c_{i_{1}}c_{i_{2}}\cdots c_{i_{n}}fdc,$$

e.g. *F* = *ρ*, *F*_*i*_ = *M*_*i*_ = *ρv*_*i*_, *F*_*kk*_ = 2*ϵ*. Often, authors decompose the moments into their irreducible parts (traces, trace-free tensors), and/or into moments of the peculiar velocity **C** [24]. This leads in particular to the specific internal energy ε, which is related to the temperature *θ*, the temperature tensor **Θ** and the heat flux vector **q**,

2.5 $$\varepsilon =\frac{3}{2}\theta =\frac{m}{2\rho }\int C^{2}fdc,\;\Theta_{ij}=\frac{m}{\rho }\int C_{i}C_{j}fdc,\;q_{i}=\frac{m}{2}\int C^{2}C_{i}fdc.$$

Note that *θ* = Θ _*kk*_/3. Moreover **P = ***ρ***Θ** is the pressure tensor, and *p* = *P*_*kk*_/3 = *ρθ* is the pressure for the ideal gas.

The entropy density of the gas is given by

2.6 $$\eta =-k_{\text{B}}\int f\ln\frac{f}{y}dc,$$

with a proper scaling constant *y*. Evaluation with the Maxwellian *f*_M_ gives the well-known equilibrium entropy density of the ideal gas

2.7 $$\eta_{E}=\frac{k_{\text{B}}}{m}\left\{\frac{1}{2}\ln\left[{\left(\frac{2\pi m^{2}}{h^{2}}\right)}^{3}\frac{m^{2}\theta^{3}}{\rho^{2}}\right]+\frac{5}{2}\right\}\rho ,$$

where *h* is Planck’s constant. We emphasize that the Boltzmann equation has full thermodynamic structure in the sense of GENERIC [14], and is Galilean invariant.

### (b) Moment equations

Thermodynamic transport theories aim at a macroscopic description of processes, that is the main interest is in the averages (moments) introduced above, and not in the details of the distribution function. Hence, one is not interested in solving the Boltzmann equation, but rather in finding, and then solving, equations for the moments themselves. Note that the macroscopic moments describe collective behaviour of the particles, while on the level of the microscopic distribution function, the individual particle behaviour is resolved.

Multiplication of the Boltzmann equation with $mc_{i_{1}}c_{i_{2}}\cdots c_{i_{n}}$, (*n* = 0, 1, 2, 3, …) and subsequent integration over the microscopic velocity results in a coupled system of moment equations of balance law form

2.8 $$\frac{\partial F_{i_{1}i_{2}\cdots i_{n}}}{\partial t}+\frac{\partial F_{i_{1}i_{2}\cdots i_{n}k}}{\partial r_{k}}=\Pi_{i_{1}i_{2}\cdots i_{n}}\;(n=0,1,2,3,\ldots ),$$

where

2.9 $$\Pi_{i_{1}i_{2}\cdots i_{n}}=m\int c_{i_{1}}c_{i_{2}}\cdots c_{i_{n}}Sdc$$

are the moments of the collision term. The resulting system of equations is infinite, since the flux under the divergence is the next higher moment, i.e. it has one index more than the variable under the time derivative. The infinite moment system is equivalent to the Boltzmann equation, and, most notably for the present context, both have the proper GENERIC structure.

### (c) Finite number of moments and Grad closure

The moment method was pioneered by Grad in 1949 [27]. In principle, the method proceeds as follows: first, one chooses a meaningful finite subset $F_{A}=\int \phi_{A}fdc$ of the $F_{i_{1}i_{2}\cdots i_{n}}$ as variables, where *A* is a multi-index counting through the chosen variables, and *ϕ*_*A*_ are corresponding tensor combinations of the microscopic velocity. The set *ϕ*_*A*_ must be chosen such that the resulting set of equations is Galilei invariant.

Special cases that will be considered below are the five moment case, where *F*^(5)^ = (*F*, *F*_*i*_, *F*_*kk*_), and the 10 moment case, where *F*^(10)^ = (*F*, *F*_*i*_, *F*_*ij*_). Note that the decomposition of the *F*_*A*_ into explicit terms with velocity and central moments leads to alternative sets of variables; e.g. for five and 10 moments one might use the variables *u*^(5)^ = (*ρ*, *v*_*i*_, *θ*) and *u*^(10)^ = (*ρ*, *v*_*i*_, Θ _*ij*_).

The corresponding moment equations, of the form

2.10 $$\frac{\partial F_{A}}{\partial t}+\frac{\partial {\bar{F}}_{Ak}}{\partial r_{k}}=\Pi_{A},$$

are not a closed system of equations for the variables *F*_*A*_, since they contain the fluxes ${\bar{F}}_{Ak}$ and the production terms Π_*A*_ (if the variable *F*_*A*_ is of the form $F_{i_{1}i_{2}\cdots i_{n}}$ then the flux ${\bar{F}}_{Ak}$ is given by $F_{i_{1}i_{2}\cdots i_{n}k}$). Closure relations, that is constitutive functions, are needed, to relate fluxes and productions to the variables.

In kinetic theory, variables, fluxes and productions are all defined as integrals over the distribution function *f*(**r**, *t*, **c**). Hence, the closure problem can be solved by construction of a distribution function that is a functional of the variables, $f_{\text{cl}}(r,t,c)=F(F_{A}(r,t),c)$ with $\int \phi_{A}f_{\text{cl}}dc=F_{A}$. This leads to constitutive equations for fluxes ${\bar{F}}_{Ak}=\int \phi_{A}c_{k}f_{\text{cl}}dc={\bar{F}}_{Ak}(F_{B}(r,t))$ and productions $\Pi_{A}=\int \phi_{A}S(f_{\text{cl}},f_{\text{cl}})dc=P_{A}(F_{B}(r,t))$ that are functionals of the variables as well. Typically, one expects constitutive relations that link e.g. the flux ${\bar{F}}_{A}(r,t)$ to the values of the variables *F*_*A*_ and some of their derivatives at the same point (**r**, *t*).

Grad [27,28] was the first to develop a closure method for moment equations with arbitrary number of variables. If the set of variables is chosen well, the resulting set of transport equations can describe gas processes outside the realm of classical hydrodynamics with sufficient accuracy, such as thermal stresses, non-Fourier energy transport, etc.

Specifically, the Grad distribution function is constructed as a perturbation of the equilibrium distribution,

2.11 $$f_{\text{Grad}}=f_{\text{M}}\left(1-\sum_{A}\lambda_{A}\phi_{A}\right),$$

with coefficients *λ*_*A*_(*F*_*B*_) determined from the linear system $F_{A}=\int \phi_{A}f_{\text{Grad}}dc$. Accordingly, the constitutive relations for fluxes and productions are purely local, ${\bar{F}}_{Ak}(F_{B}(r,t))$, Π_*A*_(*F*_*B*_(**r**, *t*)). Hence, non-local effects are accounted for through the moment equations, which form a set of first order partial differential equations.

Since the *ϕ*_*A*_ are polynomials in **c**, the Grad distribution *f*_Grad_ is not strictly positive. While the Maxwellian suppresses large values of the velocity, so that the distribution is mostly positive, it is nevertheless impossible to compute the corresponding entropy density from $-k_{\text{B}}\int f_{\text{Grad}}\ln(f_{\text{Grad}}/y)dc$. With this, the Grad-type moment equations are not accompanied by a proper entropy balance with strictly non-negative production. Not too far from equilibrium, the equations are hyperbolic, but they lose hyperbolicity for sufficiently strong deviation from equilibrium (e.g. Ruggeri determines a hyperbolicity radius around equilibrium [9]). This restricts the applicability in nonlinear processes [29].

Linearized Grad-type moment equations have proper entropy inequality, and are stable [30]. One can say that the Grad method preserves enough of the properties of the Boltzmann equation at least close to equilibrium. Even without a formal entropy inequality, the nonlinear Grad-type equations are able to describe processes outside of the hydrodynamic regime sufficiently well. However, the lack of structure—loss of 2nd law and hyperbolicity—might lead to breakdown of solutions, or unphysical results for strong non-equilibria.

### (d) Closure by entropy maximization

For the Grad closure, the distribution is a perturbation of the Maxwellian that assumes (very small) negative values at large velocities, and hence the entropy (2.6) cannot be determined. Maximization of entropy (MaxEnt) offers an alternative route to closure, that is in fact centred on entropy, and produces a positive distribution function, and a proper form of the 2nd law [31,32].

The idea is to chose the least biased distribution that is compatible with the chosen variables, which is obtained by maximizing the entropy (2.6) under the constraint of given values for the variables *F*_*A*_. Taking care of the constraints by means of Lagrange multipliers Λ_*A*_, this results in a distribution that is the exponential of a polynomial in **c**,

2.12 $$f_{\text{max }}=\exp\left[-\sum_{A}\Lambda_{A}\phi_{A}\right].$$

The thermodynamic structure for the resulting moment equations is just as for the phenomenological theory of rational extended thermodynamics, that will be discussed further below. The foundation in kinetic theory, however, provides additional insight and, more importantly, limitations.

The distribution must asymptotically vanish for large velocity (else moments are infinite), hence the highest power of **c** in the exponent must be of the form *c*^2*α*^. The set of variables must be chosen accordingly, e.g. the requirement is fulfilled for *F*^(5)^ = (*F*, *F*_*i*_, *F*_*kk*_) and *F*^(10)^ = (*F*, *F*_*i*_, *F*_*ij*_). It is not fulfilled for *F*^(13)^ = (*F*, *F*_*i*_, *F*_*ij*_, *F*_*kki*_) where the largest power is *c*^2^**c**_*i*_ which is odd in velocity space, and will lead to blow up unless the corresponding Lagrange multiplier Λ_*kki*_ vanishes. Therefore, to incorporate stress tensor *F*_*ij*_ and energy flux *F*_*kki*_ into a theory one must add at least one more variable, which leads to a theory of 14 variables with *F*^(14)^ = (*F*, *F*_*i*_, *F*_*ij*_, *F*_*kki*_, *F*_*kkll*_). Extension to other moment sets is straightforward.

Normally, one is interested in a closed form of transport equations, that is one would want to determine explicit constitutive functions ${\bar{F}}_{Ak}(F_{B})$ and Π_*A*_(*F*_*B*_). However, apart from the five and 10 moment cases, integrals of the distribution (2.12) can only be obtained numerically. Owing to the inherent difficulties, there are only few contributions that fully exploit the maximum entropy distribution [33,34], and only the five and 10 field versions are evaluated without approximations. A maximum entropy closure is highly desirable for the proper thermodynamic structure. Moreover, results for one-dimensional kinetic equations show considerable improvement over Grad closures with the same number of moments. Hence, it is quite unfortunate that the mathematical details prevent the systematic development of MaxEnt moment systems with arbitrary number of moments.

## 3. Rational extended thermodynamics

Methods of extended thermodynamics aim to describe processes far from equilibrium, which are characterized by non-local effects and history dependence. While the classical approach to include such effects is to allow higher gradients of hydrodynamic variables into constitutive equations, RET extends the space of variables by adding nonequilibrium quantities, such as stress tensor, heat flux, other fluxes and even fluxes of fluxes. The task of an extended thermodynamic framework is to develop proper evolution equations for the additional variables, so that thermodynamic structure is guaranteed.

Typically, these evolution equations are first order partial differential equations in space and time. History dependence and non-locality are accounted for through these evolution equations, and if longer history dependence, or wider non-locality, is required, one will add more nonequilibrium variables and their evolution equations. Higher order constitutive functions typically lead to problems such as instabilities, whereas the equations of extended thermodynamics have good mathematical properties.

### (a) Basic equations

RET is strongly motivated by the moment method as outlined above, but emphasizes the need of a proper entropy law for all processes. While the moment method with entropy maximization as outlined above is limited to ideal gases, RET imposes a rather similar structure, but aims at describing other materials as well. A complete description of the theory is given in the monograph by Müller & Ruggeri [9].

Starting point is the choice of a set of *N* thermodynamic variables *F*_*A*_, *A* = 1, …, *N*, for which one postulates transport equations in the balance law form (2.10). This balance law form appears to be natural when considering matter within an arbitrary volume *V*, since one would expect changes of a property *F*_*A*_ within *V* effected by either a flux ${\bar{F}}_{Ak}$ over the system boundary ∂*V*, or due to a production Π_*A*_—which can be positive or negative—within the volume. In kinetic theory, as seen above, balance law form arises naturally from taking moments of the Boltzmann equation. However, strict balance law form might be lost in model reduction methods, e.g. when elements of equations are weighted differently, and removal of some terms from the equations changes their structure. An example for this is the application of the order of magnitude method to find a condensed set of equations for hard sphere molecules in [35], where the resulting equations (written for peculiar moments) cannot be cast in balance law form.

Next, similar to the Grad and MaxEnt closures, RET demands constitutive relations which are fully local in space and time, that is the fluxes and productions at (**r**, *t*) depend only on the variables themselves at the same space–time location,

3.1 $${\bar{F}}_{Ak}(r,t)={\bar{F}}_{Ak}(F_{B}(r,t)),\;\;\Pi_{A}(r,t)=\Pi_{A}(F_{B}(r,t)).$$

The constitutive equations are restricted by the entropy principle, which postulates the existence of an entropy obeying the additional balance law

3.2 $$\frac{\partial \eta }{\partial t}+\frac{\partial \Phi_{k}}{\partial r_{k}}=\sigma \geq 0,$$

where *η*(*F*_*B*_) is the entropy density, Φ_*k*_(*F*_*B*_) is the entropy flux and *σ*(*F*_*B*_) is the entropy production. That is, also entropy flux and productions are given by local constitutive laws. The entropy production must be strictly non-negative for all thermodynamic processes, i.e. all solutions of the balance laws for the *F*_*A*_. Evaluation with Liu’s lemma [22] introduces Lagrange multipliers Λ_*A*_(*F*_*B*_) which relate entropy and variables as

3.3 $$d\eta =\Lambda_{A}dF_{A},\;d\Phi_{k}=\Lambda_{A}d{\bar{F}}_{Ak},\;\sigma =\Lambda_{A}\Pi_{A}\geq 0.$$

Accordingly,

3.4 $$\frac{\partial \eta }{\partial F_{A}}=\Lambda_{A}\;\text{and}\;\frac{\partial \Phi_{k}}{\partial {\bar{F}}_{Ak}}=\Lambda_{A}$$

and hence we have the symmetry relations

3.5 $$\frac{\partial^{2}\eta }{\partial F_{A}\partial F_{B}}=\frac{\partial \Lambda_{A}}{\partial F_{B}}=\frac{\partial \Lambda_{B}}{\partial F_{A}}\;\text{and}\;\frac{\partial^{2}\Phi_{k}}{\partial {\bar{F}}_{Ak}\partial {\bar{F}}_{Bk}}=\frac{\partial \Lambda_{A}}{\partial {\bar{F}}_{Bk}}=\frac{\partial \Lambda_{B}}{\partial {\bar{F}}_{Ak}}.$$

Moreover, RET has also the requirement of convexity,

3.6 $$-\frac{\partial^{2}\eta }{\partial F_{A}\partial F_{B}}\;\text{is positive definite}$$

(convexity as concept and name comes from mathematics, where researchers use the mathematical entropy density −*η* instead of the physical entropy density *η*). Convexity ensures that the transformation *F*_*A*_(Λ_*B*_) is invertible to Λ_*A*_(*F*_*B*_), and also is related to entropy assuming a maximum at equilibrium.

Using a Legendre transform to switch to the Lagrange multipliers as variables yields

3.7 $$\frac{\partial F_{A}}{\partial \Lambda_{B}}\frac{\partial \Lambda_{B}}{\partial t}+\frac{\partial {\bar{F}}_{Ak}}{\partial \Lambda_{B}}\frac{\partial \Lambda_{B}}{\partial r_{k}}=\Pi_{A}$$

and due to symmetry (3.5) and convexity (3.6), this is a symmetric hyperbolic system with convex extension [36]. The mathematical literature proves that systems of this kind have desirable properties, most importantly well-posedness of the initial boundary value problem. Finally we note that RET requires Galilean invariance of the equations, which leads to a clear identification of the role of velocity in the resulting equations.

The above just lists the required structure, which holds also for moment equations with entropy maximization as discussed above. The difficulty is to fill all this with meaning, by finding explicit sets of equations, for which all elements (fluxes, productions, Lagrange multipliers) are explicitly known.

While the RET structure is in principle open to arbitrary materials, most applications concern ideal gases, including quantum gases such as phonons and photons [9]. For these, entropy maximum closure in kinetic theory has the very same structure, with the Lagrange multipliers from maximization and the Liu procedure being identical [31].

We have already discussed the difficulties to perform the entropy maximum closure in kinetic theory for more than 10 moments. While the evaluation of the phenomenological RET structure proceeds quite differently from the kinetic theory approach—where one has to integrate a distribution function—it is also quite challenging, and for classical monatomic gases there is no RET system above the 10 moment case that has the full structure as outlined above. Further examples, including chemical reactions, radiation and viscoelasticity, can be found in [37].

All RET systems with 13, 14 and more moments discussed, e.g. in [9,38], are, in fact, approximations and do not have the full RET structure. Rather, these approximations are similar, if not identical, to Grad-type moment systems, with the same limitations as these, in particular only the linearized equations have an entropy, and hyperbolicity is lost for larger deviations from equilibrium states.

The equivalence between RET for the ideal gas and maximum entropy closure was never fully exploited to discuss feasibility of the macroscopic approach. For instance, a MaxEnt system with 13 variables is impossible due to blow up of the distribution function as discussed above. From this, one would guess that the full RET approach for 13 moments (or any other set with odd closure) is impossible as well. On the other hand, the (somewhat limited) success in applying the MaxEnt closure by fitting of closure relations that account for the singularities indicates that a RET closure might be possible—at least for moment sets for which the MaxEnt distribution remains integrable.

### (b) Comments

RET and MaxEnt approach have highly desirable properties, in particular the mathematical structure guarantees well-posed equations with entropy. However, only very few systems are available that have this structure, and all of these are too simple. For example, the 10 moment case discussed further below includes viscous stresses but no heat transfer.

All known systems of extended thermodynamics, or moment methods, with larger sets of variables are approximations. They might give spurious results if used outside their—not well-defined—range of applicability, or might change their mathematical properties. Nevertheless, these theories describe nonequilibrium process, with history dependence and non-localities, very well, in excellent agreement with experiments, or solutions of the full Boltzmann equation [9].

As approximations, they obviously inherit some of the structure of the stricter theories, but outside the linearized case the extent of this is not well understood. We can say that one sacrifices rigorous mathematical and thermodynamic structure for the sake of having powerful tools for the description of nonequilibrium processes.

Ideally, one would hope to find transport equations that combine accurate predictions and proper thermodynamic structure. With the strict RET and MaxEnt formalism unattainable, we will look now at GENERIC, in the hope that this general framework offers more flexibility to attain this goal.

## 4. GENERIC

The basic idea of GENERIC is to generate irreversible dynamics by the entropy, just like Hamiltonian dynamics, which is considered as the prototype of reversible dynamics, is generated by the energy. The assumption of energy conservation under irreversible dynamics is matched by assuming entropy conservation under reversible dynamics. Entropy production can arise only from irreversible processes, and the amount of entropy production can be specified in terms of a friction matrix or a dissipation potential [20,39].

### (a) Basic equations

The GENERIC framework is based on the following general equation for the nonequilibrium reversible–irreversible coupling:

4.1 $$\frac{\text{d}x}{\text{d}t}=L\frac{\delta E}{\delta x}+M\frac{\delta S}{\delta x},$$

where *x* represents a set of independent variables that allows for an autonomous description of a given nonequilibrium system. For local field theories, the total energy *E* and entropy *S* as functions of *x* are given in terms of the corresponding densities,

4.2 E=∫e dr,S=∫η dr.

The linear operators, or matrices, *L* and *M*, which can depend on *x*, are known as the Poisson and friction matrices, respectively. Equation (4.1) is supplemented by the complementary degeneracy requirements

4.3 $$L\frac{\delta S}{\delta x}=0$$

and

4.4 $$M\frac{\delta E}{\delta x}=0,$$

which are strong formulations of the conservation of entropy (energy) by reversible (irreversible) dynamics. Further general properties of *L* can be discussed most conveniently in terms of the Poisson bracket

4.5 $$\{A,B\}=\frac{\delta A}{\delta x}\cdot L\frac{\delta B}{\delta x},$$

where *A*, *B* are sufficiently regular, real-valued functions defined on the space of independent variables and the dot indicates a canonical product.

Further conditions for *L* can now be stated as the antisymmetry property

4.6 {A,B}=−{B,A},

the product or Leibniz rule

4.7 {AB,C}=A{B,C}+B{A,C},

and the Jacobi identity

4.8 {A,{B,C}}+{B,{C,A}}+{C,{A,B}}=0,

where *C* is another sufficiently regular function on the state space. These properties are well known from the Poisson brackets of classical mechanics, and they express the essence of reversible dynamics. Contrary to the case of classical mechanics, the Poisson bracket for dissipative systems must be degenerate so that the degeneracy requirement (4.3) for the entropy can be fulfilled.

The most important further requirement for the friction matrix *M* is positive-semidefiniteness. This requirement is a strong formulation of the second law of thermodynamics as it, together with the degeneracy requirement (4.3), implies the inequality

4.9 $$\frac{\text{d}S}{\text{d}t}=\frac{\delta S}{\delta x}\cdot M\frac{\delta S}{\delta x}\geq 0.$$

Whereas the Poisson matrix *L* clearly needs to be antisymmetric, the symmetry properties of *M* are less obvious. In most applications, *M* can be assumed to be symmetric. However, in view of the Onsager symmetry and Casimir antisymmetry occurring in linear irreversible thermodynamics [3–6], we expect that *M* can also be antisymmetric (a detailed discussion of the relation between the Onsager–Casimir symmetry of linear irreversible thermodynamics and the symmetry properties of the friction matrix *M* of GENERIC can be found in Section 3.2.1 of [14]). The possibility of an antisymmetric *M* has actually been used in the context of slip and of turbulence [40].

In general, the friction matrix incorporates a number of distinct dissipative processes. It is then helpful to write the friction matrix as the sum of separate contributions associated with the individual dissipative processes. If each contribution possesses the properties of positive-semidefiniteness, energy degeneracy and symmetry/antisymmetry, then also the total friction matrix is positive-semidefinite and leads to energy conservation.

### (b) Comments

An important strength of the GENERIC framework is the flexibility in the choice of variables. This flexibility is made possible by using Poisson brackets and Hamiltonians in the formulation of reversible dynamics, so that one does no longer need the guidance from balance equations to formulate reversible dynamics. Neither is one restricted to considering only convection as a reversible process. The reward of the flexibility in the choice of variables is the enormous number and variety of problems that can be and have been studied within the GENERIC framework (see Appendix E of [14] or [41] for reviews).

The Jacobi identity (4.8), which is a highly restrictive condition for formulating proper reversible dynamics, expresses the time-structure invariance of Poisson brackets [14,42]. More precisely, if two observables *A* and *B* are evolved in time, their time-dependent Poisson bracket also satisfies the evolution equation for an observable. The Jacobi identity is deeply related to the proper implementation of the symmetry properties of a system. Finally, the Jacobi identity may be considered as an integrability condition guaranteeing that the convection mechanism, or other reversible effects, can be integrated up consistently from small to large deformations.

Numerical methods that focus on the proper treatment of entropy can have significant advantages, as has been illustrated for the Lattice Boltzmann method [43,44]. In the context of 13 moment equations, it has been shown that the numerical solution of shock wave problems [45] and the formulation of boundary conditions [46] (see also [30]) can benefit enormously if the existence of an entropy is exploited properly. The search for GENERIC integrators, which preserve (as much as possible of) the GENERIC structure under time discretization in the same spirit as symplectic integrators preserve the Hamiltonian structure, is still in its beginning (see [[47], and references therein, [48]]).

## 5. Comparison

The comparison of RET and GENERIC is restricted to situations in which RET is actually applicable. Moment equations are hence the natural choice for our comparison. As both approaches rely on the existence of an entropy functional, they both have the problem of expressing the entropy density in terms of moment variables. This can be done most naturally by introducing particle distribution functions parametrized by the moments, as discussed in the context of closures in §2c. A strategy for the successful construction of entropies for moment equations, which is based on considering general rather than only Cartesian vector and tensor transformation behaviour, has been developed in [49–52]. The close relationship between the Lagrange multipliers of RET and the entropy gradients of GENERIC should be noted.

The most challenging problem in applying GENERIC often is the identification of a valid Poisson bracket to represent the reversible contribution to dynamics. In the case of moment equations, the involvement of velocities in all moments makes it quite hopeless to find a Poisson bracket that expresses the convection mechanism implied by the Boltzmann equation. As the strategy for overcoming this problem is the main innovation of the present paper, we discuss some general ideas for describing convection before we enter a more detailed comparison. The matching of relaxation terms turns out to be a relatively simple exercise because there is much less thermodynamic structure in them.

### (a) Convection mechanism

The convection mechanism expressed by the left-hand side of the balance equations (2.8) is neither of the upper convected nor of the lower convected type frequently used in rheological modelling, for both of which it is known that they can readily be expressed by means of well-known Poisson brackets (see Section 2.3.1 of [14]). The discrepancy looks minor but is crucial: either there is a sign problem or the role of the velocity gradient tensor and its transpose is interchanged. These differences keep us from finding a Poisson bracket even for the simplest case of 10 moments, where anisotropic Gaussians are the perfect trial functions for the particle distribution function (contrary to what was said on p. 309 of [14]).

The novel strategy that we propose in this paper is to shuffle the deviations from an admissible convection mechanism from the reversible to the irreversible contribution of GENERIC. Of course, these irreversible ‘convective corrections’ should not produce entropy and should hence be realized through an antisymmetric contribution to the friction matrix, a contribution associated with Casimir symmetry.

For vectors in position space, it is natural to be convected by an upper convected mechanism, whereas the lower convected mechanism is natural for momentum vectors. As we are dealing with moments of the momentum distribution, we begin with the Poisson bracket providing lower convected derivatives. We then realize that the deviation from the Boltzmann-type convection mechanism can be described in terms of the vorticity (this simplicity would not arise if we had started from upper convected derivatives). The details of this construction are next elaborated for the 10 moment equations. It can be generalized in a straightforward way to the 13 moment equations (as we shall elaborate elsewhere).

### (b) 10 moment equations

As seen from RET, or entropy maximization, the 10 moment equations in full balance law form for the variables *ρ*, *ρv*_*i*_, *ρ*(*v*_*i*_*v*_*j*_ + Θ _*ij*_) read

5.1 $$\left.\begin{array}{rl} & \frac{\partial \rho }{\partial t}+\frac{\partial \rho v_{k}}{\partial r_{k}}=0,\\ & \frac{\partial \rho v_{i}}{\partial t}+\frac{\partial \rho (v_{i}v_{k}+\Theta_{ik})}{\partial r_{k}}=0\\ \text{and}\;\; & \frac{\partial \rho (v_{i}v_{j}+\Theta_{ij})}{\partial t}+\frac{\partial \rho (v_{i}v_{j}v_{k}+3\Theta_{(ij}v_{k)})}{\partial r_{k}}=-\frac{\rho }{\tau }(\Theta_{ij}-\theta \delta_{ij}), \end{array}\right\}$$

where the production term arises from the Boltzmann equation for Maxwell molecules, or from the Bhatnagar–Gross–Krook model for the collision term in the Boltzmann equation, and the round brackets around indices indicates symmetrization in these indices. The first two equations are continuity and momentum balance equations, and the third is the evolution equation for momentum flux. The energy balance is included as the trace of the last equation. If one sets Θ _*ij*_ = *θδ*_*ij*_ and considers only the trace of the last equation the above reduce to the Euler equations for an ideal gas, which is the five moment version of RET.

For studying the above equations within GENERIC, we express the 10 moment equations in terms of *ρ*, **M** = *ρ***v**, **Θ**, where they become

5.2 $$\begin{array}{rl} \frac{\partial \rho }{\partial t} & =-\frac{\partial }{\partial r}\cdot (v\rho ), \end{array}$$

5.3 $$\begin{array}{rl} \frac{\partial M}{\partial t} & =-\frac{\partial }{\partial r}\cdot (vM+\rho \;\Theta ) \end{array}$$

5.4 $$\begin{array}{rl} \text{and}\;\frac{\partial \Theta }{\partial t} & =-v\cdot \frac{\partial }{\partial r}\Theta -\kappa \cdot \Theta -\Theta \cdot \kappa^{T}-\frac{1}{\tau }(\Theta -\theta \;1). \end{array}$$

Here, we have introduced the velocity gradient tensor with components *κ*_*jk*_ = ∂*v*_*j*_/∂*r*_*k*_. In the second-moment equation (5.4), we can readily distinguish between convection terms (involving **v** or **κ**) and relaxation terms (involving the relaxation time *τ*). Note that the trace of **Θ**, as a thermodynamic variable, is exempt from relaxation.

For an ideal gas, the energy is generally given in terms of zeroth, first and second moments, so that we always have

5.5 $$E=\int_{V}\left(\frac{M^{2}}{2\rho }+\frac{\rho }{2}\text{tr}\;\Theta \right)d^{3}r.$$

The case of 10 moments is particularly simple because the maximum entropy principle leads to an anisotropic Gaussian probability distribution in momentum space. The corresponding entropy is given by the following generalization of (2.7),

5.6 $$S=\frac{k_{\text{B}}}{m}\int_{V}\left\{\frac{1}{2}\ln\left[{\left(\frac{2\pi m^{2}}{h^{2}}\right)}^{3}\frac{m^{2}}{\rho^{2}}\det\Theta \right]+\frac{5}{2}\right\}\rho \;d^{3}r.$$

These expressions for the energy and entropy lead to the gradients

5.7 $$\frac{\delta E}{\delta x}=\left(\begin{array}{c} \frac{3}{2}\theta -\frac{1}{2}v^{2}\\ v\\ \frac{1}{2}\rho 1 \end{array}\right),\;\frac{\delta S}{\delta x}=\left(\begin{array}{c} \frac{\eta }{\rho }-\frac{k_{\text{B}}}{m}\\ 0\\ \frac{1}{2}\rho \frac{k_{\text{B}}}{m}\;\Theta^{-1} \end{array}\right),$$

where *η* is the entropy density, that is, the integrand in (5.6).

From the perspective of RET, the 10 moment equations have a simple and natural form. We next consider the problem from the perspective of GENERIC. As already mentioned in the previous section, if we express the convection mechanism in (5.4) in terms of a Poisson operator *L*, we are faced with an unpleasant surprise: it does not satisfy the Jacobi identity. The Jacobi identity allows only two options for the convection of tensors, which are associated with upper and lower convected time derivatives (see Sect. 2.3.1 of [14] for a detailed discussion). Differences of position vectors are naturally associated with upper convective behaviour (any type of flow or deformation implies transformations for all the points occupied by a material [53,54], and hence also for all the vectors connecting two points), whereas lower convected behaviour is the natural choice for momentum vectors (this is the complementary choice in the sense that the contraction of a pair of vectors with upper and lower convective behaviour is convected as a scalar). To prepare a valid GENERIC formulation, we rewrite the second-moment equation (5.4) in the form

5.8 $$\frac{\partial \Theta }{\partial t}=-v\cdot \frac{\partial }{\partial r}\Theta -\kappa^{T}\cdot \Theta -\Theta \cdot \kappa +\omega \cdot \Theta -\Theta \cdot \omega -\frac{1}{\tau }(\Theta -\theta \;1),$$

where **ω** = **κ**^*T*^ − **κ** is the vorticity tensor. The terms involving **v** or **κ** now correspond to a lower convected derivative so that the corresponding Poisson operator in index notation,

5.9 $$L=-\left(\begin{array}{ccc} 0\; & \frac{\partial }{\partial r_{k}}\rho \; & 0\;\\ \rho \frac{\partial }{\partial r_{i}}\; & \frac{\partial }{\partial r_{k}}M_{i}+M_{k}\frac{\partial }{\partial r_{i}}\; & -\frac{\partial \Theta_{kl}}{\partial r_{i}}+2\frac{\partial }{\partial r_{(k}}\Theta_{l)i}\;\\ 0\; & \frac{\partial \Theta_{ij}}{\partial r_{k}}+2\Theta_{k(i}\frac{\partial }{\partial r_{j)}}\; & 0\; \end{array}\right),$$

is known to satisfy the Jacobi identity (see Table 2.1 of [14]). We use the convention that the indices *i*, *j* refer to matrix multiplications from the left side, whereas *k*, *l* refer to matrix multiplications from the right side.

Our next goal is to represent the terms involving **ω** in (5.8), which describe the mismatch between moment convection and lower convective behaviour, as an irreversible contribution to GENERIC. We construct an antisymmetric matrix *M*^cc^ (‘cc’ refers to convective corrections) because we do not expect these convective effects to be associated with entropy production. The choice

5.10 $$M^{\text{cc}}=\frac{2m}{k_{\text{B}}}\left(\begin{array}{ccc} 0 & 0 & 0\\ 0 & 0 & \frac{\partial }{\partial r_{s}}\theta [\delta_{i(k}\Theta_{l)s}-\Theta_{i(k}\delta_{l)s}]\\ 0 & \theta [\Theta_{s(i}\delta_{j)k}-\delta_{s(i}\Theta_{j)k}]\frac{\partial }{\partial r_{s}} & \frac{1}{3\rho }({\left[\omega ,\Theta \right]}_{ij}\Theta_{kl}-\Theta_{ij}{\left[\omega ,\Theta \right]}_{kl}) \end{array}\right),$$

with the commutator [**ω**, **Θ**] = **ω** · **Θ** − **Θ** · **ω**, (i) provides the desired modification of (5.8), (ii) leaves the mass and momentum balances unchanged, (iii) implies energy degeneracy as a strong formulation of energy conservation, and (iv) does not cause any contribution to entropy production.

Finally, the relaxation term involving *τ* in (5.8) needs to be reproduced. As the derivative of the entropy (5.6) is proportional to **Θ**^−1^, this requires a contribution *M*^relax^ to the *M* matrix that is quadratic in **Θ**. The following symmetric, positive-definite, and energy conserving contribution to the friction matrix reproduces the desired linear relaxation term,

5.11 $$M^{\text{relax}}=\frac{2m}{3\tau k_{\text{B}}\rho }\;\frac{1}{1-(1/9)\;\text{tr}\Theta \;\text{tr}\Theta^{-1}}\left(\begin{array}{ccc} 0 & 0 & 0\\ 0 & 0 & 0\\ 0 & 0 & \left(\Theta_{ij}-\theta \delta_{ij})(\Theta_{kl}-\theta \delta_{kl}\right) \end{array}\right).$$

Note that, in our physically guided formulation, linear relaxation is not produced by a quadratic entropy and a constant contribution to the *M* matrix. As the denominator of the prefactor in (5.11) is somewhat artificial, it might actually be more natural to consider nonlinear relaxation mechanisms.

For the 10 moment equations, we find perfect agreement between RET and GENERIC. As mentioned before, the full RET structure cannot be achieved for 13 or 14 moments. It can be shown, however, that the matrices *M*^cc^ and *M*^relax^ can be generalized to the case of 13 moments. Further important terms in the 13 moment equations, in particular those required for reproducing Fourier’s law for the heat flux in the limit of fast relaxation, can be incorporated by an additional antisymmetric contribution to the friction matrix. The generalization of the entropy to the 13 moment case has been discussed in [49–51]. The details of the GENERIC formulation of 13 moment equations will be presented elsewhere.

### (c) Regularized moment equations

Most extended theories lead to first order partial differential equations which form a hyperbolic system (in the range of applicability). Hyperbolic systems have finite speed of propagation of disturbances, and in the early days of RET, these finite speeds were touted as describing proper physics [8,9], since infinite speeds of propagation should be impossible. However, the Boltzmann equation for classical particles allows infinite particle speeds, hence infinite speed of heat transfer as described through Fourier’s law is not in contradiction. Indeed, the finite propagation speeds in RET systems are artefacts of the theories, and not a picture of actual physics.

Processes in which these finite speeds play a significant role, e.g. the resolution of shocks, pose a significant challenge, due to the occurrence of unphysical sub-shocks [55]. These sub-shocks are not encountered for regularized moment equations, which effectively replace the strictly local closure such that fluxes and productions depend at most on first order space derivatives of the variables *F*_*A*_. Detailed accounts of the methods used to regularize a Grad-moment system can be found in [24,56,57]. Effectively, the regularizing terms are remnants of higher order moment equations, which are reduced by means of scaling arguments based on Knudsen number.

The thermodynamic structure, or the lack thereof, of the regularized moment equations is similar to the underlying Grad models: a proper second law exists only for the linear case [30], but the equations are highly meaningful also for nonlinear settings, including shocks, where they produce smooth structures [58]. An entropy law for the regularized 13-moment equations in the nonlinear case is possible after adding non-trivial nonlinear terms [52].

From the perspective of GENERIC, regularization can readily be implemented as a dissipative process by means of a symmetric friction matrix that contains two partial derivatives. This type of friction matrices is very common in the modelling of transport processes where second-order partial differential equations are ubiquitous.

## 6. Conclusion and challenges

We have demonstrated that RET is a special case of GENERIC for the special choice of variables inspired by the moment equations obtained from the Boltzmann equation for rarefied gases. This is not only true for the infinite moment hierarchy, which is equivalent to the Boltzmann equation, but also for properly truncated moment equations. In general, the GENERIC closures are different from the ones considered in the literature. The usefulness of these new truncations remains to be explored, most importantly, for the 13 moment equations.

The essential new step in recognizing 10 (or 13) moment equations as GENERIC is to allow for irreversible but non-entropy-producing contributions to convection. In the context of linear irreversible thermodynamics, this option is associated with Casimir symmetry, which is perfectly natural. These complications in the convection mechanism arise because all variables are related to moments of velocity rather than conformational properties. There might exist a deep analogy between moment equations and the problem of turbulence. The non-entropy-producing but irreversible energy cascade in turbulence is related to vortices in the velocity on different length scales. In the moment hierarchy, we have a number of velocity moments, which cover couplings and relaxation phenomena over a range of different scales. In both cases, all variables are related to velocities so that the convection mechanism becomes extremely involved. Both cases involve vorticity in a crucial way. Details of this analogy remain to be elaborated.

The fundamental difference between RET and GENERIC lies in the flexibility in the choice of variables. Simple recipes for finding a list of good variables, in terms of which a given problem of interest can be described by autonomous evolution equations, may seem appealing. However, flexibility in choosing the variables and the understanding associated with the choice of variables are essential features of modern nonequilibrium thermodynamics. Considering its roots, it is natural that the main area of application of RET is the theory of gases. The application of RET to rarefied and dense gases is the topic of the article by Arima, Ruggeri and Sugiyama in this theme issue. One should note, however, that the first 50 years of rheology have been ruled by differential constitutive equations for the stress tensor, which is the momentum flux. By adding the heat flux, even nonisothermal rheology can be based on flux variables. Also the equations for relativistic hydrodynamics are usually based on flux variables [59–66] (see also article by Romenski, Peshkov, Dumbser and Fambri in this theme issue).

If the ideas of the present work are generalized to relativistic gases, this suggests the natural implication that the hydrodynamics of relativistic gases is fundamentally different from the hydrodynamics of relativistic liquids (in the nonrelativistic case, the universal form of hydrodynamics for all kinds of fluids is dictated by conservation laws). The hydrodynamics of gases requires the presence of Casimir-type irreversible convection, whereas the hydrodynamics of liquids does not require that [62,63]. The convection mechanism for liquids is simpler than for gases because the transport properties result from interactions in real space (even after equilibration in momentum space). These observations remain to be elaborated in detail.

## Data accessibility

This article has no additional data.
